# 

**Published:** 1867-12

**Authors:** 


### THE
### IOWA MEDICAL JOURNAL,

### A BI-MONTHLY.

Price $2 00 per annum, in advance. If not paid before the issue of the second No., the price will be $3 00.

---

*Gentlemen of the Profession:*

Owing to the financial embarrassment of 1857–8, the IOWA MEDICAL JOURNAL, which had completed its fourth volume, was suspended for the want of material aid. The unsettled condition of the country since then, and my absence in Europe the last year, has prevented our embarking in the enterprise. As the county is safe, and the profession are again heartily engaged in the active duties of civil life, I propose, should I receive the necessary encouragement from my professional brethren of my own and the adjoining States, to continue what I have now commenced—the publication of the fifth volume of the IOWA MEDICAL JOURNAL. For the present, it will be a bi-monthly, of 32 pages—not so large as formerly, but costing the publisher more. Its size will be increased as rapidly as the proceeds will justify; and we hope, with the aid of our professional friends, to make the IOWA MEDICAL JOURNAL a welcome visitor to every lover of Medical Science in the West.

Send us your $2 00 at once. This encourages the printer, and is the pocket argument.

Next in importance is your contributions, which will always be acceptible, if short and to the point, and more particularly if they contain facts, and common sense.

All letters and communications for the JOURNAL may be addressed to

J. C. HUGHES, M. D.,

Keokuk, Iowa.

OUR ADVERTISEMENTS.—We would call the attention of the Profession, and all others interested, to the cards of our citizens who have so liberally aided us in this, the 1st No. of our JOURNAL. They are all men of business and integrity, and are prepared to accommodate the Profession and public with everything in their line upon the most reasonable terms.

We take pleasure in saying, that no city of the West offers greater inducements for the purchase of books, medicines or instruments, than the city of Keokuk. The houses of M. W. Westcott and Ed. S. Carter & Co., can furnish you with everything in the line of books and stationery........The Messrs. Kerr & Fuller, F. H. O'Connor, Christfield & Co., John T. Wilkinson, and Wilkinson, Bartlett & Co., can do up, in the most approved style, either in original packages, by prescription, or at wholesale and retail, everything in the way of medicines, instruments, or anatomical preparations.......Our friend S. G. Bridges is always on hand with the useful as well as ornamental, in the way of jewelry; but that which interests the Profession especially, is, his fine assortment of spectacles suited to every variety of defective vision, where artificial lenses are required........Messrs. Tepfer and Vermillion are getlemen who know how to keep hotel. Pleasure seekers, wishing a pleasant home and retreat for the Summer months, could not do better than to come to Keokuk, stopping at the Deming or Tepfer House, where they can have every wish gratified.

